# The spectrum of sepsis-associated encephalopathy: a clinical perspective

**DOI:** 10.1186/s13054-023-04655-8

**Published:** 2023-10-05

**Authors:** Romain Sonneville, Sarah Benghanem, Lina Jeantin, Etienne de Montmollin, Marc Doman, Augustin Gaudemer, Michael Thy, Jean-François Timsit

**Affiliations:** 1 INSERM UMR 1137, Université Paris Cité, 75018 Paris, France; 2 Department of Intensive Care Medicine, Bichat-Claude Bernard University Hospital, APHP, 46 Rue Henri Huchard, 75877 Paris Cedex, France; 3 Department of Intensive Care Medicine, Cochin University Hospital, APHP, 75014 Paris, France; 4 Department of Neurology, Rothschild Foundation, Paris, France; 5 Department Radiology, Bichat-Claude Bernard University Hospital, APHP, 75018 Paris, France

**Keywords:** Encephalopathy, Delirium, Coma, Sepsis, Seizures, Prognosis

## Abstract

Sepsis-associated encephalopathy is a severe neurologic syndrome characterized by a diffuse dysfunction of the brain caused by sepsis. This review provides a concise overview of diagnostic tools and management strategies for SAE at the acute phase and in the long term. Early recognition and diagnosis of SAE are crucial for effective management. Because neurologic evaluation can be confounded by several factors in the intensive care unit setting, a multimodal approach is warranted for diagnosis and management. Diagnostic tools commonly employed include clinical evaluation, metabolic tests, electroencephalography, and neuroimaging in selected cases. The usefulness of blood biomarkers of brain injury for diagnosis remains limited. Clinical evaluation involves assessing the patient's mental status, motor responses, brainstem reflexes, and presence of abnormal movements. Electroencephalography can rule out non-convulsive seizures and help detect several patterns of various severity such as generalized slowing, epileptiform discharges, and triphasic waves. In patients with acute encephalopathy, the diagnostic value of non-contrast computed tomography is limited. In septic patients with persistent encephalopathy, seizures, and/or focal signs, magnetic resonance imaging detects brain injury in more than 50% of cases, mainly cerebrovascular complications, and white matter changes. Timely identification and treatment of the underlying infection are paramount, along with effective control of systemic factors that may contribute to secondary brain injury. Upon admission to the ICU, maintaining appropriate levels of oxygenation, blood pressure, and metabolic balance is crucial. Throughout the ICU stay, it is important to be mindful of the potential neurotoxic effects associated with specific medications like midazolam and cefepime, and to closely monitor patients for non-convulsive seizures. The potential efficacy of targeted neurocritical care during the acute phase in optimizing patient outcomes deserves to be further investigated. Sepsis-associated encephalopathy may lead to permanent neurologic sequelae. Seizures occurring in the acute phase increase the susceptibility to long-term epilepsy. Extended ICU stays and the presence of sepsis-associated encephalopathy are linked to functional disability and neuropsychological sequelae, underscoring the necessity for long-term surveillance in the comprehensive care of septic patients.

## Introduction

Sepsis-associated encephalopathy (SAE) is a severe neurologic syndrome characterized by a diffuse dysfunction of the brain caused by sepsis, a life-threatening condition resulting from the dysregulated response of the body to an infection. At the acute phase, patients with SAE typically present with an acute onset of encephalopathy, ranging from delirium to coma [1]. The pathophysiology of sepsis-associated encephalopathy is complex and involves multiple mechanisms that collectively contribute to brain dysfunction and injury [2]. One of the primary mechanisms is the release of pro-inflammatory cytokines, which leads to the disruption of the blood–brain barrier (BBB), causing an influx of immune cells and inflammatory mediators into the brain. This inflammation triggers the activation of microglia, the immune cells of the brain, which further release cytokines and reactive oxygen species, leading to oxidative stress and neuronal damage. Other important acute phase mechanisms they notably include cerebral hypoxia, metabolic disturbances, microvascular and BBB alterations, and neurotransmitter imbalances. SAE can be possibly triggered or aggravated by secondary causes, including systemic insults, renal or hepatic dysfunction, environmental factors, and the use of neurotoxic agents. Although SAE is classically seen as a fully reversible pathophysiological process due to systemic inflammation, there is increasing evidence suggesting that sepsis may be associated with structural brain injury and neurologic sequelae in the long term [3, 4].

In the present article, we review recent findings in the field of SAE focusing on its epidemiology, diagnosis, and management at the acute phase. We also provide an update on long-term effects of SAE observed in sepsis survivors.

## Epidemiology and short-term outcomes

SAE is most frequently defined as an acute encephalopathy occurring during sepsis or septic shock, and not attributable to any other cause than sepsis itself [5]. SAE is thought to be the most common cause of encephalopathy in the intensive care unit (ICU) [6]. In a landmark study conducted in 50 non-sedated ICU septic patients, SAE, defined by a score on the Glasgow coma scale (GCS) < 15 was observed in 54% of patients [7]. In the most recent cohorts, where SAE was defined as sepsis associated with a GCS < 15 or delirium features, reported incidences were 53% in a French ICU multicenter cohort [8], and up to 68% in a cohort of septic patients from United Sates databases MIMIC-IV and eICU [9]. In a recent large multicenter study, sepsis-associated delirium had a median duration among affected participants of 3 (interquartile range 2–6) days [10]. With these definitions, however, SAE remain a broad syndrome with severity ranging from mild delirium to deep coma, impacting patient prognosis accordingly. It has been well demonstrated that occurrence of SAE is independently associated with short-term mortality [7–9]. Severity of SAE is also correlated to mortality, patients with GCS 3 to 8 having the worst prognosis (HR 3.37, 95% CI 2.82–4.03) [8]. Interestingly, even mild alterations of mental status, defined by a score on the GCS of 13 or 14 are independently associated with an increased risk of death (HR 1.38, 95% CI 1.09–1.38).

## Risk factors and clinical presentation

Most of the available data on acute encephalopathy in the ICU has been generated from studies conducted in the general population. Few specific epidemiological studies have been conducted in patients with sepsis, and risk factors for SAE identified in these studies are described in Table 1 [7, 8, 11, 12]. These studies are biased by the lack of consensual definitions for SAE and the use of different sepsis criteria. Therefore, large multicenter epidemiological studies specific to SAE are needed.

**Table 1:** Risk factors for sepsis-associated encephalopathy

	Medical history	On ICU admission	During ICU stay
Non-modifiable factors	Older age Chronic kidney disease Chronic alcohol abuse History of neurologic disease History of cognitive impairment Long-term use of psychoactive drugs	Medical admission Mechanical ventilation Acute renal failure Bacteremia *Staphylococcus aureus* infection	Bacteremia
Modifiable factors	–	Hypoglycemia < 3 mmol/l Hyperglycemia > 10 mmol/l Hypercapnia > 45 mmHg Hypernatremia > 145 mmol/l	Midazolam Cefepime

Clinical evaluation of SAE is challenging in the ICU because neurologic assessment can be confounded by several factors, including fever, metabolic derangements, and the use of hypnotic agents in mechanically ventilated patients. SAE manifests as a rapid change from baseline cognitive status or level of consciousness, and presents with a wide range of symptoms, from mild delirium (19%) to coma (40%) [1, 8]. Coma or hypoactive delirium is the most common presentation of SAE, whereas agitation (_~_10% of cases) and dysautonomia are less frequent [8]. Convulsive seizures (_~_2% of cases) and focal signs (_~_1% of cases) are uncommon and should trigger investigations to rule out brain injury. Thus, underdiagnosis of SAE is probable in the absence of systematic screening with validated tools [13]. Conversely, SAE may be the first sign of early sepsis, and any new-onset encephalopathy must prompt clinicians to screen their patients for infection.

## Neuroimaging

Data on the usefulness of brain CT studies in patients with SAE is limited. In a meta-analysis conducted in adults with acute non-traumatic encephalopathy, CT abnormal findings were observed in 11% of cases [14]. In medical ICU patients, most common acute findings diagnosed from non-contrast head CT studies included infarction (5% of cases) and hemorrhage (4% of cases) [15]. In patients presenting with acute coma or any other clinical sign suggesting brainstem involvement, angio-CT should be performed to rule out acute basilary artery occlusion. Acute basilary occlusion may represent up to 10% of unexplained non-traumatic coma, with more than 40% of cases misdiagnosed with non-contrast head CT [16].

Brain magnetic resonance imaging (MRI) is indicated in presence of focal signs, brainstem symptoms, new-onset seizures, and in case of persistent encephalopathy in the absence of common confounders (i.e. metabolic/toxic factors and sedation). It is recommended to include a diffusion-weighted imaging (DWI) sequence in the MRI protocol, which is the most sensitive sequence for detection of cerebral ischemia and inflammatory changes. MRI alterations diagnosed at the acute phase of SAE include parenchymal lesions and atrophy, that are reported in about 55% and 16% of cases, respectively [17, 18]. Ischemic lesions are diagnosed in 14–27% of SAE patients presenting persistent encephalopathy, focal signs or seizures during ICU stay [17, 19]. Infarct patterns can be multiple (67%), large (43%), and/or junctional (29%) and are independently associated with disseminated intravascular coagulation and lower platelet counts [20]. Ischemic lesions result from both macrocirculatory compromise like low blood pressure and disrupted autoregulation of cerebral blood flow, as well as microcirculatory changes such as damaged blood vessel linings and increased blood clotting. These factors collectively lead to cerebrovascular damage.

White matter lesions (WML) are observed in a significant percentage (14–81%) of SAE patients presenting with persistent encephalopathy [19, 21]. These lesions share a periventricular distribution pattern similar to the brain changes seen in small vessel disease linked to hypertension. Posterior reversible encephalopathy syndrome is reported in 9% of SAE cases [21]. WML are associated with vasogenic edema, and are mostly located in the superior frontal sulci or parieto-occipital sulci.

Brain atrophy is more pronounced in patient with SAE than in healthy controls [18]. This atrophy appears to be diffuse, with a significant reduction in the total volumes of the cerebral cortex, white matter, and hippocampus [22]. Cerebral atrophy is more pronounced in patients with higher APACHE II and SOFA scores and is associated with worse neurologic outcomes [17].

Acute neuroimaging changes have prognostic significance as predictors of disability and survival in the first year following SAE. In a single-center study, ischemic stroke was found to be independently associated with increased ICU mortality and poor functional status at 6 months [20]. In another study, acute neuroimaging abnormalities (i.e., infarction, hemorrhage and/or edema) were independently associated with ICU mortality, 1-year mortality following ICU discharge, and disability. Greater brain atrophy has been correlated with worse cognitive performance at 12 months [23]. In a recent study, the presence of PRES was not associated with worse outcomes [21].

## Electroencephalography and evoked potentials

### EEG

**Fig. 1:**
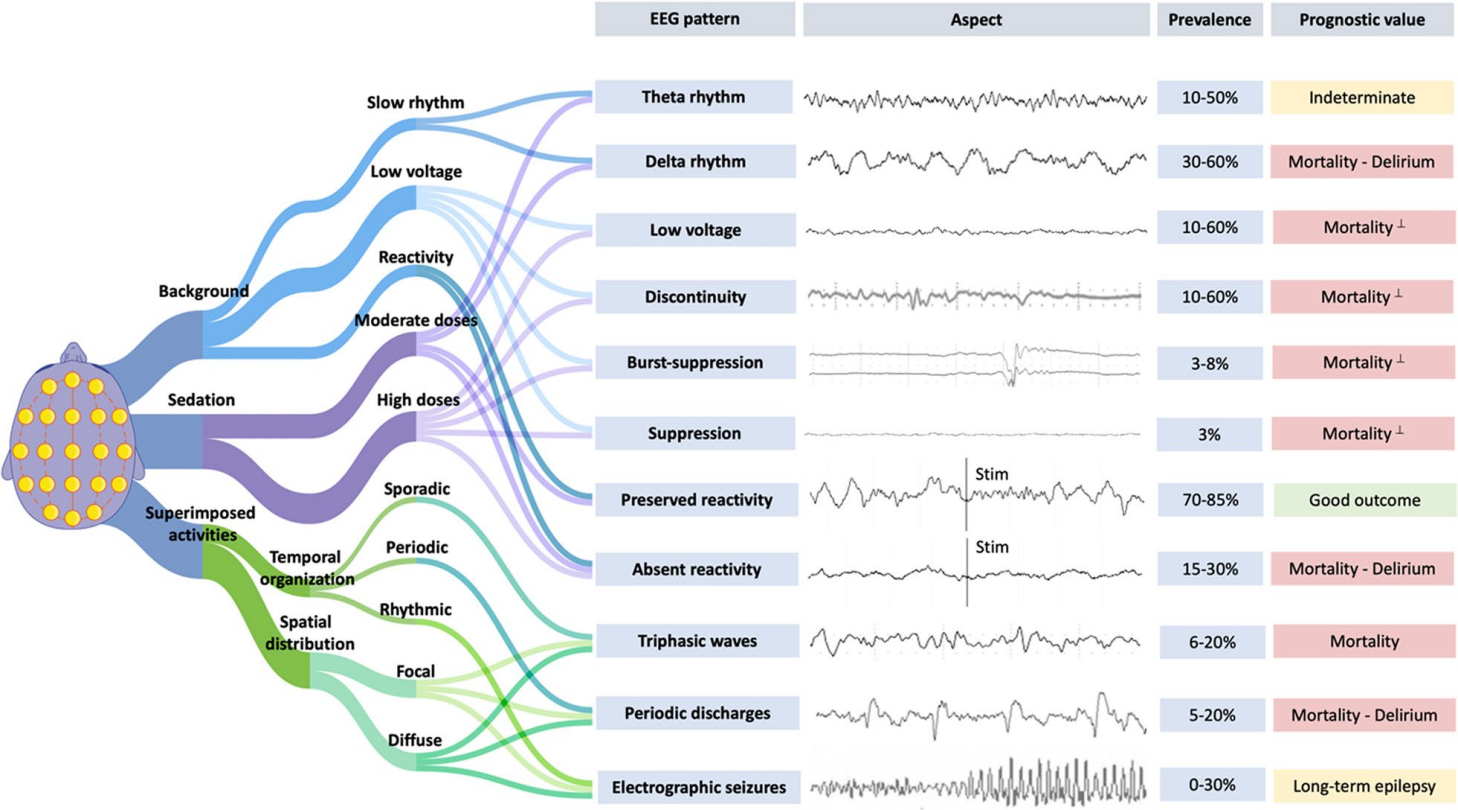
Aspect, prevalence, and prognostic value of EEG patterns in sepsis-associated encephalopathy. (Prevalence and prognosis data from Azabou et al. [31], Berisavac et al. [32], Gilmore et al. [36], Hosokawa et al. [30], Benghanem et al. [37], Velissaris et al. [28]). ^⊥^These EEG patterns observed without concomitant sedation were associated with mortality. Definitions: Background frequency is described as δ (0.2–3.5 Hz), θ (4–7.5 Hz), α (8–13 Hz) or β (14–30 Hz) bands. Low voltage (200ms). Sporadic triphasic waves: rare slow wave with an initial negative deflection (upward) followed by a positive component (downward) and then negative again; when associated to encephalopathy, they are ample diffuse slow waves, frequently prominent in the fronto-central regions. Periodic discharges: abundant periodic abnormalities (spike or wave, with a return to the EEG background between abnormalities), during >50% of the recording. Rhythmic discharges: abundant rhythmic discharges (spike or wave, without return to the EEG background between abnormalities) during >50% of the recording. Electrographic seizure: rhythmic discharges at >2.5 Hz for ≥10 seconds or any pattern with definite spatio-temporal evolution and lasting ≥10 seconds

EEG can be a valuable tool in the positive diagnosis of SAE, for excluding non-convulsive status epilepticus, and for prognostication (Fig. 1). In non-sedated patients, EEG is indicated in patients with altered mental status (ranging from delirium to coma), seizures or stereotyped abnormal movements (especially myoclonus). In mechanically ventilated patients, EEG recordings can be obtained if there is delayed awakening, typically in case of persistent unresponsiveness after 48 h after sedation discontinuation [24].

One of the main challenges is the impact of sedation on EEG background, which is dependent on the dosage and the specific sedative used. Sedatives can lead to a dose-dependent slowing of the EEG background. Benzodiazepines often produce diffuse rapid rhythms (> 13Hz), whereas propofol and barbiturates may result in low voltage, discontinuous patterns at moderate doses, and burst suppression or suppression patterns at higher doses [25, 26] (Fig. 1).

EEG is sensitive but not specific for assessing SAE, as similar abnormal patterns can appear in different encephalopathies. So, understanding the patient's clinical situation and considering other possible diagnoses is vital when interpreting EEG results. Moreover, there is variability in how neurophysiologists interpret EEG and the American Clinical Neurophysiology Society (ACNS) introduced specific terms for critical care EEG to address this issue [27].

Early EEG abnormalities may precede clinical neurologic impairment and correlate with the severity of encephalopathy [28, 29]. In recent studies, EEG background is described as slow with a theta or delta dominant rhythm in 10 to 50% and 30 to 60% of patients, respectively (Fig. 1) [28, 30–33]. Amplitude and continuity can also be affected, from low voltage and discontinuous background (10–60% of cases) to burst suppression or suppression (3–8% of cases) [31, 32, 34]. The more severe EEG patterns are mostly observed in sedated patients, and thus the proportion of EEG abnormalities related to sedation versus SAE is not easy to assess. Diffuse triphasic waves are observed in 6–20% patients [30–32], and periodic discharges are reported in 5–20% patients [31, 32]. The discrepancies in the prevalence of EEG abnormalities between studies could be explained by the type/duration of EEG recording (standard vs continuous), the time course and severity of sepsis, and the lack of standardization in interpretation.

The pathophysiology of electrographic seizures (ESz) in SAE remains debated, but could be related to the increased neuronal excitotoxicity and epileptogenic factors, including neurotoxic antimicrobials, metabolic disturbances, and severe acute kidney injury. Most ESz are non-convulsive, highlighting the interest of continuous EEG recording [35]. ESz are observed in 0–30% of cases [31, 32, 34, 36].

Some EEG patterns are associated with delirium, including slow delta rhythm, absence of EEG reactivity, discontinuity, and presence/burden periodic discharges (PDs) [31, 34, 37]. PDs may contribute to brain hypoxia and might be considered as a cause of secondary brain injury [34]. Conversely, rapid beta activity is associated with a reduced risk of delirium [38].

Some EEG patterns are also associated with ICU mortality. The absence of reactivity has been shown to be independently associated with ICU and 1 year mortality [31, 32, 36]. A recent prospective study highlighted that triphasic waves, slow delta background and suppressed EEG were the most frequent patterns observed within 24h prior to death [32]. The score on the Synek scale, which was developed in anoxic and trauma patients, is also associated with mortality in septic patients, similarly to PDs presence/burden [31, 34]. In a general population of medical ICU patients remaining unresponsive after sedation interruption, a pattern consisting of a reactive standard electroencephalography with a background frequency greater than 4 Hz was associated with reduced mortality [24]. It is important to highlight that the capacity of these EEG patterns to predict delirium or mortality is only moderate on an individual patient level.

### Evoked potentials

Evoked potentials (EPs) are neural responses time-locked to some stimulus and differ from EEG signals as they are stimulus-induced. EPs reflect the combined activity of many neurons firing together and necessitate averaging multiple sensory or auditory stimulations. These components are labeled based on their polarity (negative as "N" and positive as "P") and their latency (measured in milliseconds) from the stimulus. [39]. Somato-sensory evoked potential (SSEP) are the most commonly used EP in the ICU, mostly for neuroprognostication, and the bilateral absence of N20 is recognized as the most robust marker of poor outcome in comatose patients after cardiac arrest [40]. Other types of EPs include as brainstem auditory evoked potentials (BAEPs) and long latency event-related potentials (ERPs) with mismatch negativity (MMN) and P300 responses [41]. The interest of EPs for the diagnostic and prognostic of SAE remains debated. A prospective cohort of septic patients suggested that subcortical (i.e., N20–N23 interlatency) and cortical (N20–N70 interlatency) pathways of SSEP were impaired in 34% and 84% of patients, respectively, these late latencies being correlated with the APACHE III score [42]. The intracranial conduction time (ICCT, namely P14–N20 latency) assessed by SSEP and the intrapontine conduction time (IPCT) assessed by BAEP could be interesting makers in predicting ICU mortality and delirium, in deeply sedated critically ill patients. One study suggested that ICCT impairment was associated with ICU mortality (OR 2.69, 95%CI 1.05–6.85), and that IPCT was only delayed in delirious patients. These ICCT and IPCT impairments could be considered as early indicators of brain and brainstem dysfunction [43]. In deeply sedated critically ill patients, a greater MMN amplitude was observed in patients who awakened compared to those who did not [44]. The utilization of EPs is hindered by several factors, including a restricted availability of devices in the ICU setting, difficulties in interpretation, and a moderate prognostic value in the sepsis population.

## Blood biomarkers

Blood biomarkers associated with neuronal injury, specifically neuron-specific enolase (NSE) and neurofilament light (NfL), as well as biomarkers linked to glial injury, such as protein S100 beta (PS100), were evaluated in sepsis patients to anticipate the onset of SAE and predict outcomes. NSE is the most accessible biomarker, and the prevalence of elevated NSE levels (i.e. > 12.5µg/L) in sepsis varies between 28 and 53% [45, 46]. Previous studies showed a modest increase in NSE concentrations during sepsis, with median serum levels of 6.6 [IQR 4.1–13.8] µg/L [45], 18.8 [IQR 13.9 –30.5] µg/L [34], and 30.33 [IQR 19.6–46.5] µg/L [47].

In a single-center study, a NSE threshold > 24.15µg/L (AUC 0.66) had a specificity of 83% and a sensitivity of 54.2% for the diagnosis of SAE [48]. One prospective cohort study found no correlation between NSE levels and mortality at day 28 [46]. Conversely, two prospective cohorts found an association between NSE levels and ICU or hospital mortality (NSE at day 4 > 25.94µg/L, AUC 0.75 [47]; NSE > 24.15µg/L, AUC 0.59 [48]). Another retrospective study demonstrated that abnormal NSE levels on ICU admission were associated with a 23.3% risk of death, and each doubling of NSE level was linked to a 7.3% increased risk of death [45]. One study suggested that NSE levels > 12.5µg/L were associated with a 29.3% risk of delirium, and each doubling of NSE level was associated with an additional 5.2% risk of delirium [5].

The prognostic value of glial injury biomarkers was examined in several studies, highlighting conflicting results. Previous prospective cohort studies indicated that high PS100 levels were associated with hospital mortality (PS100 > 0.131 μg/L, AUC 0.73) [48], but also with hypoactive delirium [46]. However, two other studies did not find any association between PS100 levels and outcomes, including occurrence of SAE and altered cognition in the long term [49, 50].

One study investigated the link between serum NfL levels and SAE outcomes [19]. Among sepsis patients, serum NfL levels increased over time, contrasting with stable levels in non-sepsis patients. Notably, SAE patients had notably higher plasma NfL values, and these values were connected to the severity of SAE. Elevated plasma NfL levels were also associated with poorer long-term functional outcomes.

One prospective study found that serum concentrations of Glial Fibrillary Acidic Protein (GFAP), a protein expressed by astrocytes, were higher in SAE patients compared to non-SAE patients [51]. Serum GFAP concentrations > 0.536 ng/ml predicted mortality (AUC 0.77), and higher GFAP levels were associated with worse long-term outcomes. Serum concentrations of microRNAs (mRNAs) have also been considered as diagnostic and prognostic biomarkers of SAE, although their use in clinical practice remains limited [52]. Of note, biomarker profiles differ between patients with SAE, sepsis, and delirium, implying that the underlying pathways associated with SAE are distinct from those associated with delirium and sepsis [53].

Using blood biomarkers for SAE diagnosis faces challenges due to undefined optimal assessment timing, uncertainty about the precise SAE onset, inconclusive findings about prognostic benefits and definitive thresholds, and limited access to certain biomarkers, impeding their routine clinical use. The profile of biomarkers differs between SAE, sepsis, and delirium patients, suggesting that pathways related to SAE are different from those related to delirium and sepsis itself.

## Management

### General measures

**Fig. 2:**
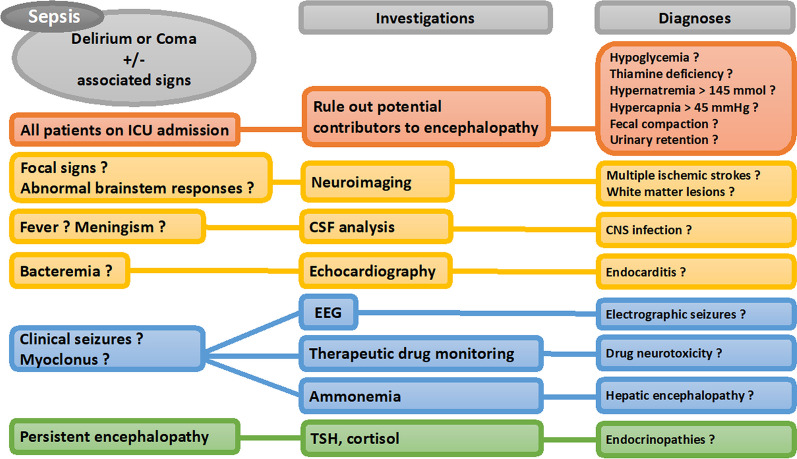
A simplified algorithm for the diagnosis of sepsis-associated encephalopathy. CSF cerebrospinal fluid; EEG Electroencephalography; ICU Intensive Care Unit; TSH thyroid-stimulating hormone

Multidisciplinary care is frequently necessary for the comprehensive management of SAE. Prompt identification and treatment of the infection are vital, typically involving the administration of antibiotics and supportive measures such as fluid resuscitation and vasopressors. Moreover, it is essential to control factors contributing to secondary brain injury, which includes maintaining adequate levels of oxygenation and blood pressure, addressing metabolic imbalances, and detecting/treating seizures. Delirium prevention is of paramount importance and implementation of the ABCDEF bundle is associated with improved survival and a reduction in the number of days of delirium and coma [54]. A simplified algorithm for the diagnosis of SAE is presented in Fig. 2.

**Fig. 3:**
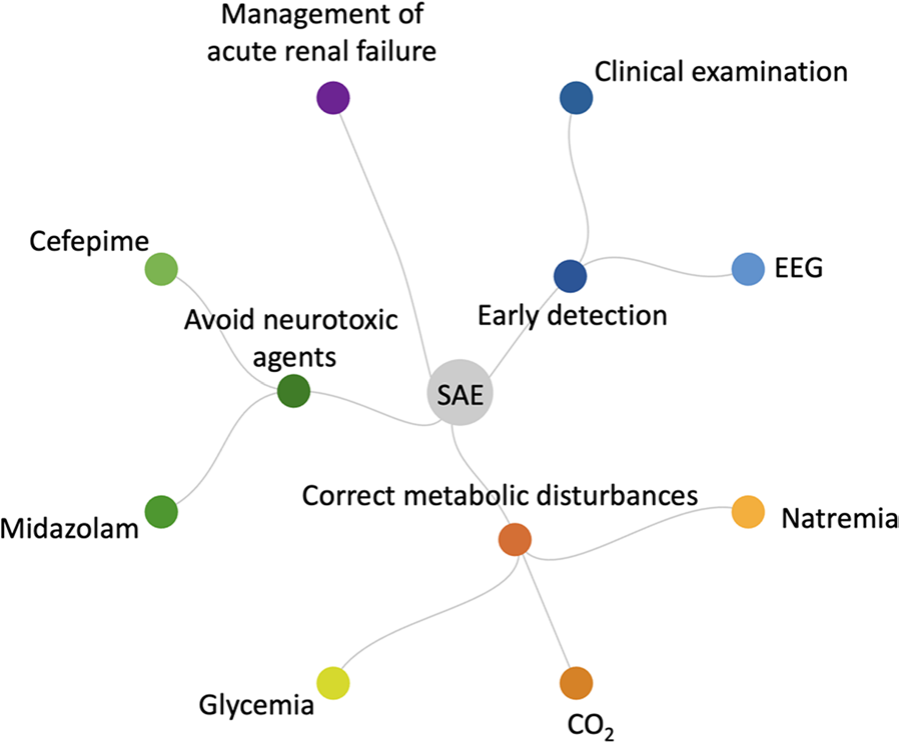
A multimodal approach for the management of sepsis-associated encephalopathy. EEG Electroencephalography; SAE Sepsis-Associated Encephalopathy

To prevent secondary insults resulting from agitation, or dysautonomia in severe cases, the use of sedatives (propofol, dexmedetomidine) or antipsychotics may be necessary. In two randomized controlled trials conducted in patients with sepsis, the use of dexmedetomidine compared to standard sedation did not result in lower rates of delirium or coma [55, 56]. The administration of midazolam should be avoided due to its independent association with encephalopathy [57–59]. Renal failure can contribute to the accumulation of various substances, including antimicrobials and hypnotics. Therefore, it is crucial to systematically monitor serum concentrations of drugs with potential neurotoxicity (such as beta-lactam antibiotics, calcineurin inhibitors, and antifungals) [60]. A multimodal approach for the management of SAE is presented in Fig. 3.

### Systemic causes of secondary brain injury

Systemic causes of secondary brain injury are frequent at sepsis onset and are linked to poorer outcomes [8]. These causes encompass hypo- and hyperglycemia, hypercapnia, and hypernatremia, as identified in prior studies [8]. However, special consideration should be given to hypoxemia and hyperoxia, anemia, hypothermia, and hyperthermia, as the failure to manage these factors adequately could theoretically result in additional brain injury. Current sepsis guidelines do not provide specific recommendations for managing systemic causes of secondary brain injury [61]. We propose that targeted neurocritical care should align with the recommendations outlined in the international sepsis guidelines [61], the post resuscitation care guidelines [40] and the neurocritical care guidelines for brain injury [62]. Proposed targets are described in Table 2.

**Table 2:** Proposed targets for control of systemic causes of secondary brain injury

Variable	Proposed target	Comments
MAP	65–80 mmHg	A higher MAP target (≥ 80mmHg) is not associated with reduced mortality [61, 63] A higher MAP target is associated with higher RASS scores during ICU stay [64]
PaO_2_	80–120 mmHg	Hyperoxia is associated with increased mortality [65]
PaCO_2_	35–45 mmHg	Hypercapnia (> 45 mmHg) is associated with an increased risk of SAE [8]
Temperature	36–38.3°C	Fever (> 38.4 °C) is associated with higher mortality [66, 67]
Natremia	135–145 mmol/L	Hypernatremia is associated with an increased risk of SAE [8]
Glycemia	5–10 mmol/L	Hypoglycemia (< 3 mmol/l) and hyperglycemia (> 10 mmol/l) are associated with an increased risk of SAE [8]
Hemoglobin	> 7g/dL	A higher transfusion threshold (> 9g/dL) is not associated with decreased mortality [68, 69]

MAP mean arterial pressure; RASS Richmond agitation sedation scale; SAE Sepsis-associated encephalopathy

### Antibiotics

Three distinct phenotypes of antibiotic-associated encephalopathy have been identified: First, an acute-onset encephalopathy commonly accompanied by clinical seizure (mostly stereotyped clonus or myoclonus) or non-epileptic myoclonus, typically manifesting within days of antibiotic administration (commonly associated with cephalosporins and penicillin); second, an encephalopathy characterized by psychosis that arises within days of antibiotic administration (commonly associated with quinolones, macrolides, and procaine penicillin); and third, a subacute encephalopathy linked to cerebellar signs and MRI abnormalities that develop weeks after initiating antibiotic therapy (commonly associated with metronidazole) [70]. The most frequently observed EEG abnormalities include non-specific signs of encephalopathy, such as diffuse slowing, and generalized PDs displaying triphasic morphology.

Cefepime remains the most frequently reported molecule associated with neurologic events, with renal dysfunction being the primary risk factor for cefepime-induced neurotoxicity [71–73]. The median time for the development of neurotoxicity after initiating cefepime is 4 days. Patients commonly present with altered mental status (93%), myoclonus (37%), and/or non-convulsive seizures (28%). If neurotoxicity is suspected, serum cefepime concentration should be monitored and EEG should be systematically performed to rule out ESz, and to assess for PDs/triphasic waves. A trough serum cefepime level exceeding 20 mg/L increases the risk of neurotoxicity. Symptoms usually improve with dose reduction or discontinuation of cefepime, with a median time to improvement of 3 days. In a retrospective study, neurotoxic side effects were not observed when the trough concentration of cefepime was below 7.7 mg/L [72]. In contrast, neurologic adverse events were always present when levels exceeded 38.1 mg/L.

## Long term outcomes and recovery

**Fig. 4:**
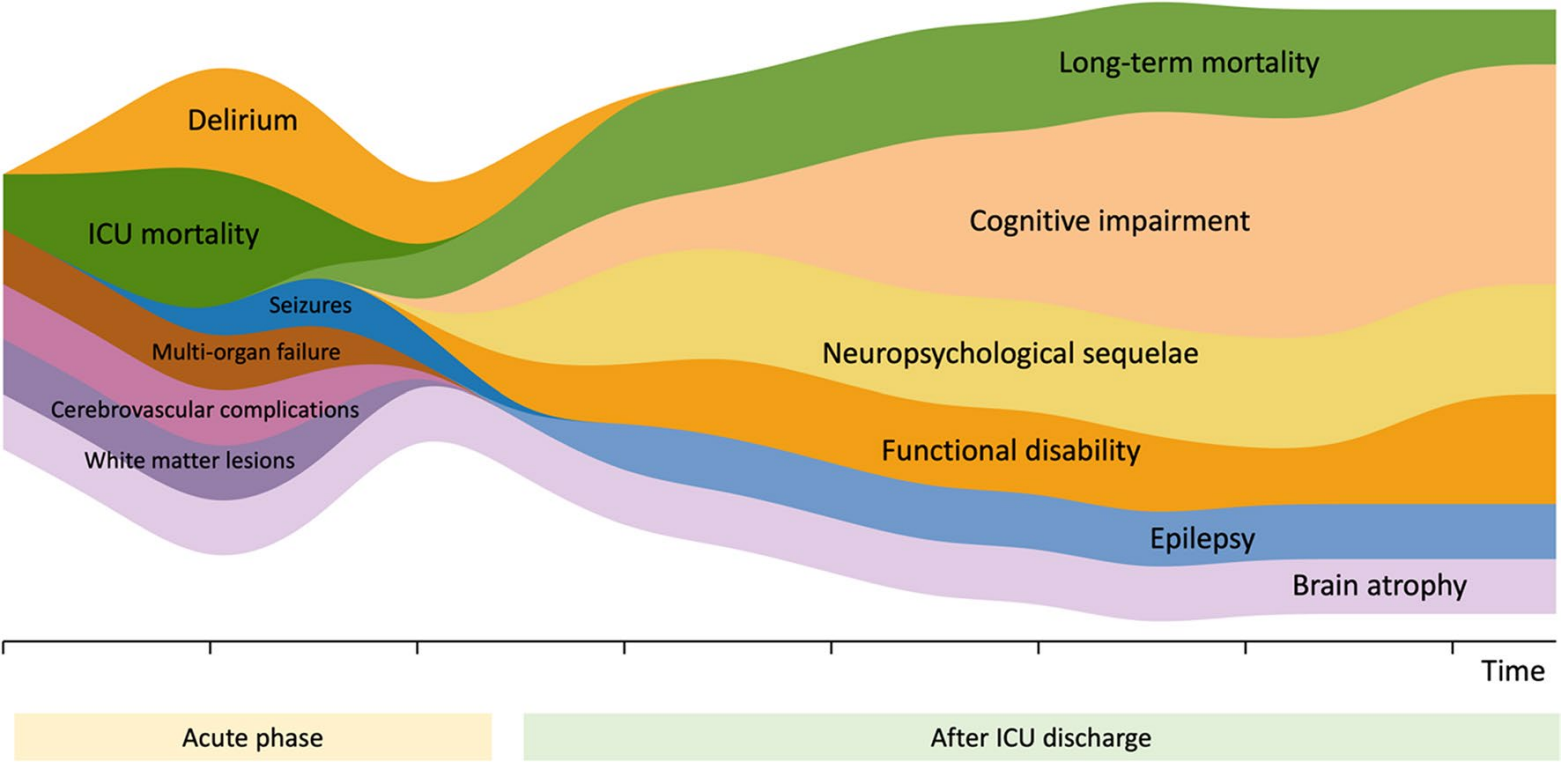
Complications associated with sepsis-associated encephalopathy at the acute phase and in the long term

Although most studies on long term outcomes have focused on the general ICU population, current data suggest that sepsis survivors experience a wide range of cognitive, psychiatric, physical, and social impairment after ICU discharge [74]. A general description is provided in Fig. 4. In a secondary analysis of international randomized trials, one third of adults with sepsis had died after six months and one third were no longer able to perform daily living activities [75].

### Cognitive impairment

Inflammatory processes are thought to participate in early brain alterations but also in long-term cognitive impairment [76]. In a prospective cohort of patients followed during nine years, sepsis episodes were associated with an increased risk of developing dementia [77]. A multicenter prospective cohort study found that a longer duration of sepsis-associated delirium was associated with altered cognitive function at 3 and 12 months [10]. Moreover, hippocampal atrophy has been described in sepsis survivors with cognitive impairment [33]. A systematic review found CNS infection, length of hospitalization and depressive symptoms to be risk factors for post-sepsis cognitive impairment [78], while data from a large randomized controlled trial highlighted older age, longer ICU stay and mechanical ventilation to be associated with a higher risk of cognitive alterations [79]. There is controversial evidence from general critically ill and septic patients that blood biomarkers could predict cognitive impairment. In general ICU patients, IL-6 and IL-10 levels were associated with poorer cognitive performances after ICU discharge [80], but acute phase plasmatic inflammation and coagulation markers did not appear to be good predictors of cognitive dysfunction [81]. One report highlighted the association of higher E-selectin and S100B levels with worse global cognition at 3 and 12 months after respiratory failure or shock [82]. High serum levels of NSE and interferon-γ were associated with poor cognitive performance after ICU discharge [78].

### Seizures and epilepsy

Sepsis survivors face a higher long-term risk of seizures than other hospitalized patients. In a large cohort, the annual incidence of seizure after sepsis was 1.29%, with incidence rate ratios of 4.98 and 4.33, compared to the general population and hospitalized patients without sepsis, respectively [83]. Among sepsis survivors, younger patients and those with chronic kidney disease appear to be at higher risk of epilepsy [84]. Taken together, these findings suggest may be an unrecognized epilepsy risk factor leading to permanent neurologic sequelae.

### Neuropsychological consequences

Sepsis survivors experience long-term emotional and behavioral changes, including depressive symptoms, anxiety and post-traumatic stress disorder (PTSD) [2]. A study of sepsis survivors found that 12% of patients had PTSD at 6 months after ICU discharge, often with delayed onset [85]. After ICU discharge, the severity of depressive symptoms was found to be associated with chronic pain or post-traumatic stress [86]. Patients were also found to experience anxiety, fatigue, and sleep disturbance. Providing primary care interventions for 12 months to sepsis survivors after ICU discharge reduced PTSD symptoms, but did not improve psychic quality of life compared to usual care [87].

### Functional disability

Compared to mechanically ventilated patients of similar acuity and length of stay without sepsis, patients with sepsis have an increased risk of mortality and a similar risk of new disability at 6 months [88]. Critically ill patients with sepsis have higher healthcare resource use and costs but similar survival and health-related quality of life compared to matched patients without sepsis [89].

ICU-acquired weakness is another frequent complication associated with sepsis resulting from alterations of small nerve fibers [90]. Typical presentation include fatigue, muscle weakness, during ICU stay or after discharge. ICU-acquired weakness likely represents an additional indicator of long term morbidity and mortality [91].

## Conclusion

SAE is a complex condition that requires a multidisciplinary approach for its diagnosis and management. Timely identification and treatment of the underlying infection are paramount, along with effective control of systemic factors that may contribute to secondary brain injury. Upon admission to the ICU, maintaining appropriate levels of oxygenation, blood pressure, and metabolic balance is crucial. Throughout the ICU stay, it is important to be mindful of the potential neurotoxic effects associated with specific medications like midazolam and cefepime, and to closely monitor patients for non-convulsive seizures. A multimodal approach based on clinical evaluation, neuroimaging and bedside available non-invasive tools may have important prognostic implications both at the acute phase and in the long term. The potential efficacy of targeted neurocritical care during the acute phase in optimizing patient outcomes deserves to be further investigated.

SAE may lead to permanent neurologic sequelae. Seizures occurring in the acute phase increase the susceptibility to long-term epilepsy. Extended ICU stays and the presence of sepsis-associated encephalopathy are linked to functional disability and neuropsychological sequelae, underscoring the necessity for long-term surveillance in the comprehensive care of septic patients.

## Data Availability

Not applicable.

## Abbreviations

BAEP: Brainstem auditory evoked potentials
BBB: Blood brain barrier
CT: Computed tomography
DWI: Diffusion weighted imaging
EEG: Electroencephalogram
EPs: Evoked potentials
ERPs: Event related potentials
ESz: Electrographic seizures
GFAP: Glial fibrillary acidic protein
PDs: Periodic discharges
ICU: Intensive care unit
ICCT: Intracranial conduction time
IPCT: Intrapontine conduction time
MMN: Mismatch negativity
MRI: Magnetic resonance imaging
Nfl: Neuro-filament light
NSE: Neuron serum enolase
PS100: Protein S100
PTSD: Post traumatic stress disorder
SAE: Sepsis-associated encephalopathy
WML: White matter lesions
